# Metabolic engineering of *Deinococcus radiodurans* for pinene production from glycerol

**DOI:** 10.1186/s12934-021-01674-4

**Published:** 2021-09-26

**Authors:** Seyed Hossein Helalat, Carsten Jers, Mandana Bebahani, Hassan Mohabatkar, Ivan Mijakovic

**Affiliations:** 1 Biological Science and Technology, Isfahan University, Isafahan, Iran; 2 The Novo Nordisk Foundation Center for Biosustainability, Technical University of Denmark, Lyngby, Denmark; 3 Systems and Synthetic Biology Division, Department of Biology and Biological Engineering, Chalmers University of Technology, Gothenburg, Sweden

**Keywords:** Pinene, Biofuel, *Deinococcus radiodurans* R1, Metabolic engineering, Monoterpene, Glycerol

## Abstract

**Background:**

The objective of this work was to engineer *Deinococcus radiodurans* R1 as a microbial cell factory for the production of pinene, a monoterpene molecule prominently used for the production of fragrances, pharmaceutical products, and jet engine biofuels. Our objective was to produce pinene from glycerol, an abundant by-product of various industries.

**Results:**

To enable pinene production in *D. radiodurans*, we expressed the pinene synthase from *Abies grandis*, the geranyl pyrophosphate (GPP) synthase from *Escherichia coli*, and overexpressed the native 1-deoxy-d-xylulose 5-phosphate synthase. Further, we disrupted the deinoxanthin pathway competing for the substrate GPP by either inactivating the gene *dr0862*, encoding phytoene synthase, or substituting the native GPP synthase with that of *E. coli*. These manipulations resulted in a *D. radiodurans* strain capable of producing 3.2 ± 0.2 mg/L pinene in a minimal medium supplemented with glycerol, with a yield of 0.13 ± 0.04 mg/g glycerol in shake flask cultures. Additionally, our results indicated a higher tolerance of *D. radiodurans* towards pinene as compared to *E. coli*.

**Conclusions:**

In this study, we successfully engineered the extremophile bacterium *D. radiodurans* to produce pinene. This is the first study demonstrating the use of *D. radiodurans* as a cell factory for the production of terpenoid molecules. Besides, its high resistance to pinene makes *D. radiodurans* a suitable host for further engineering efforts to increase pinene titer as well as a candidate for the production of the other terpenoid molecules.

**Supplementary Information:**

The online version contains supplementary material available at 10.1186/s12934-021-01674-4.

## Background

In recent years, issues regarding sustainable development of the human society have come to the fore. Concerns regarding fossil fuels are on the rise, related to depletion of their reserves, and risks connected to their distribution and environmental impact [1, 2]. The use of fossil fuels has been related to production of greenhouse gases and augmentation of metal particles, NOX, and SOX in the atmosphere [3]. Recent reports have indicated that about 87% of global CO_2_ emissions are caused by human activities. According to current predictions, the human population will exceed 9 billion by 2050, which leads to increased concerns about sustainable development [4]. These challenges have led to a substantial push to secure energy and chemicals from sustainable sources. In this respect, microbial cell factories are playing a major role. They are capable of converting low value by-products of agriculture or industries into high value chemicals or biofuels [5].

Glycerol is a by-product of various large-scale industrial processes, such as production of soap (saponification of triglycerides) and biodiesel (transesterification). Hence, it has been proposed as a cheap carbon source for fermentation [6]. Compared to glucose, glycerol is a more reduced carbon source, delivering more reducing equivalents per unit price [7]. Hence, this polyol compound is a convenient carbon source for producing valuable molecules using microbial cell factories.

Ethanol is the most popular biofuel globally [8]. However, requests for fuels with a higher combustion power that can be applied directly in the existing engines have led to development of other compounds as advanced biofuels [9]. Butanol [10], butene and its oligomers [11], fatty acids, hydro-processed esters [12], bisabolene, farnesene [13], and pinene are examples of such advanced biofuels. Pinene stands out among these molecules as a compound that can produce a high energy level close to that of JP-10 fuel required for jet engines [14]. This monoterpene is produced in low amounts by certain plants, but pinene extraction from these plants is inefficient, tedious, and costly. In addition to its use as an advanced biofuel, pinene is widely used in the production of insecticides, fragrances, flavors, and pharmaceutical products [15].

The enzyme pinene synthase catalyzes the formation of either α- and/or β-pinene by cyclization of GPP. Pinene synthase is mostly found in plants, notably pine trees such as *A. grandis*, *Pinus taeda*, and *Picea abies*. Several pinene synthases have been characterized, with some producing both isomers of pinene (α and β), and others only one isomer. Both α and β isomers can be exploited in dimer form as a powerful biofuel (α-pinene dimer: 146.900 BTU, β-pinene dimer: 146.500 BTU) [14, 16]. Various pinene synthases have been exploited for pinene production in microorganisms. In a comparative study, the pinene synthase from *A. grandis* was shown to allow production of the highest amounts of both isomers in *E. coli* [16].

Recent advances in the field of metabolic engineering of microbial cell factories have opened a new venue for pinene production using cheap carbon sources. Most of the efforts to produce pinene in microbial cell factories have been focused on *Escherichia coli*. However, the pinene yields and titers in engineered *E. coli* strains are still below the threshold for industrial production. The highest pinene titer reported so far is 166.5 mg/L in shake flask cultures and 0.97 g/L in fed-batch fermentation [15, 17]. According to previous studies, limitations in pinene production by *E. coli* might be associated with the toxicity of pinene and GPP (the main precursor of pinene), as well as limited availability of manganese as the main cofactor of pinene synthase [16]. Given the limitations of *E. coli* for pinene production, we decided to investigate *Deinococcus radiodurans* as a potential alternative in this regard.

*Deinococcus radiodurans* is known to be extremely tolerant to different sources of stress: ionizing radiation, reactive oxygen species, UV light, heavy metals, dryness, high salinity, and alcohols. Additionally, it is able to utilize various carbon sources (different sugars, glycerol, fats, and proteins) [18, 19], making it a promising candidate for various purposes such as bioremediation, decomposition of toxic compounds, and production of valuable molecules [20, 21]. Considering that manganese is the main cofactor of most pinene synthases, including that of *A. grandis*, the presence of high levels of manganese in *D. radiodurans* (0.2–4 mM) is another positive feature [22]. Most importantly, *D. radiodurans* harbors a robust non-mevalonate GPP production pathway [23] and can produce significant amounts of the pigment deinoxanthin for which GPP is a precursor [24]. These distinguishing features make *D. radiodurans* a promising platform for producing terpene compounds in different standard or harsh conditions and growing on various carbon sources. On the other hand, some challenges such as the lack of genetic engineering tools such as strong-induciable promoters and insufficient metabolic knowledge for this strain should be considered.

In this study, *D. radiodurans* a hyperextremophile bacterium, was successfully engineered to produce pinene in a minimal medium supplemented with glycerol. Our production strain expressed the pinene synthase from *A. grandis*, the GPP synthase from *E. coli*, and overexpressed the native 1-deoxy-d-xylulose 5-phosphate synthase (DXS) in a mutant of *dr0862* (encoding phytoene synthase). This strain was capable of producing 3.2 ± 0.2 mg/L pinene in a minimal glycerol medium.

## Materials and methods

### Bacterial strains and growth conditions

For plasmid propagation, *E. coli* NM522 was grown in LB culture medium (10 g/L tryptone, 5 g/L NaCl, and 5 g/L yeast extract) at 37 °C with 250 rpm shaking. For pinene production, *E. coli* was grown in a semi-defined medium (10 g/L peptone, 5 g/L yeast extract, 5 g/L glycerol, 2 g/L NaCl, 0.15 g/L MnCl_2_, 0.5 mM MgCl_2_, and 20 mM phosphate buffer pH 7.0) at 30 °C shaking (180 rpm). *D. radiodurans* R1 was grown in PGY broth (5 g/L peptone, 2 g/L glucose, 5 g/L yeast extract, and 1 g/L K_2_HPO_4_) shaking (180 rpm) at 30 °C and on TGY agar (5 g/L tryptone, 1 g/L glucose, 5 g/L yeast extract, 1 g/L K_2_HPO_4_, 15 g/L agar) at 30 °C. To evaluate *D. radiodurans* growth and pinene production with glycerol as a carbon source, first, at 30 °C with shaking (180 rpm), we used a rich medium composed of 10 g/L peptone, 5 g/L yeast extract, 20 µM MnCl_2_, 1 mM MgCl_2_, and 0.18 mM CaCl_2_. This medium was supplemented with either 5 g/L glycerol, 5 g/L of glucose, or a combination of 2.5 g/L of both of these carbon sources. For evaluating pinene production of engineered strains, the rich medium containing 5 g/L glycerol was used in shake flasks. Then, a minimal medium with various concentrations of glycerol as the sole carbon source (10, 15 or 25 g/L glycerol, 50 mg/L cysteine, 25 mg/L histidine, 25 mg/L methionine, 1% BME vitamin mix, 20 µM MnCl_2_, 1 mM MgCl_2_, 0.18 mM CaCl_2,_ 10 g/L Na_2_HPO_4_, 2 g/L KH_2_PO_4_) was made, and the best strains were grown in 30 °C shaking (100 rpm) in shake flasks to produce pinene from glycerol as the main carbon source.. Also, different agitation speeds from 70 to 250 rpm were tested in the rich and minimal glycerol medium. When necessary, appropriate antibiotics (100 µg/ml ampicillin, 50 µg/ml kanamycin, and 10 µg/ml tetracycline for *E. coli*; 3 µg/ml chloramphenicol and 25 µg/ml kanamycin for *D. radiodurans*) were added to the medium.

### DNA manipulations and strain construction

The 1785 bp coding sequence for the *A. grandis* pinene synthase was obtained from [NCBI, Accession number: AAK83564, without signal peptide], codon-optimized for expression in *D. radiodurans*, and synthesized by GenScript. The codon-optimized gene sequence is shown in Additional file 1: Fig. S1. The synthesized gene (denoted *ps*_*Dr*_), with added *Xho*I/*Sac*I/*Nde*I restriction sites upstream of the gene, was subcloned into the plasmid pRADN1 [25] between *Xho*I and *Bam*HI sites. We decided to place the pinene synthase gene under the control of three well-known and strong promoters P_*katA*_, P_*groE*_, and P_*tufA*_ often used for constitutive gene expression in *D. radiodurans* [26]. The three promoter fragments (including their associated ribosomal binding sites), were PCR-amplified using *D. radiodurans* R1 genomic DNA as a template. All primer sequences are listed in Additional file 1: Table S1. The promoter fragments P_*katA*_ and P_*groE*_ were inserted between *Xho*I and *Ndel*I and P_*tufA*_ between *Xho*I and *Sac*I, to yield expression vectors pRAD-kP, pRAD-gP, pRAD-tP. To make plasmids for expressing a pinene synthase-GFP fusion, we first constructed vectors with the pinene synthase gene devoid of the stop codon, essentially as described above. Next, we PCR amplified *gfp* using the pCDH 513B vector as a template and inserted it between *Bam*HI and *Hind*III.

Expression of the *E. coli ispA*, encoding GPP synthase, was placed under the control of P_*katA*_. First, the promoter fragments were PCR-amplified and inserted in pRAD-kP between *Bam*HI and *Xba*I sites. Then *ispA* was PCR-amplified from *E. coli* NM522 genomic DNA and inserted the resulting fragment between *Xba*I and *Hind*III to yield the vector pRAD-P-I. Additionally, the P_*katA*_ and *ispA* fragments were inserted in pRADN1 to obtain pRAD-I. For over-expression of the native *D. radiodurans* gene *dxs* encoding 1-deoxy-D-xylulose-5-phosphate synthase, we amplified the P_*katA*_ promoter fragment and inserted it between *Hind*III and *Sal*I in pRAD-P-I. The *dxs* gene was PCR amplified using *D. radiodurans* R1 genomic DNA as a template and inserted between *Sal*I and *Sda*I to yield the vector pRAD-P-I-D. To construct pRAD-P-D, the plasmid pRAD-P-I-D was restricted with *Bam*HI and *Hind*III to remove *ispA*, treated with S1 nuclease (ThermoFisher) to remove ssDNA overhangs, and then re-ligated to yield pRAD-P-D.

It has previously been suggested that a translational fusion of pinene synthase and GPP synthase can improve the catalytic activity in pinene production [16]. To test this, we made two versions of the pinene synthase-GPP synthase fusion: one with pinene synthase in the N- terminus (Pinene synthase-linker-GPP synthase) and one with GPP synthase in the N-terminus (GPP synthase-linker-pinene synthase). In both cases, the enzymes were separated by a flexible GGGGS linker peptide. To construct the plasmids expressing these fusions, the *ps*_*Dr*_ and *ispA* genes were PCR amplified, restricted with *Sal*I/*Sac*I and *Sac*I/*Bam*HI, respectively, and inserted between *Sal*I and *Bam*HI in pRAD-P-I-D to yield pRAD-P:G-D. The plasmid pRAD-G:P-D was made analogously.

The genes *dr0862* (*crtB*) and *dr1395* encoding phytoene synthase and GPP synthase, respectively, where inactivated in *D. radiodurans* R1. This was done by replacing the genes with a kanamycin resistance gene transcribed from the P_*katA*_ promoter. To do so, we PCR amplified the up-and downstream regions of the genes as well as the P_*katA*_ promoter using *D. radiodurans* R1 genomic DNA as a template. The kanamycin resistance gene was PCR amplified using the pET-26b plasmid as the template. Next, the fragments were inserted sequentially in pET-26b (for the purpose of assembling the fragments), and finally, the full construct was used as the template for PCR with primer sets dr0862_UP fwd/ dr0862_DWN rev and dr1395_UP fwd/dr1395_Dwn rev, respectively. The PCR products were purified and used to transform *D. radiodurans* R1 (transformation described below). When inactivating *dr1395*, the PCR fragment was mixed with plasmids containing the *ispA* gene (pRAD-I and pRAD-P-I-D). In the absence of *ispA* no transformants were obtained. As both mutations were envisioned to disrupt pigment production in *D. radiodurans*, transformants without pigment production phenotype were selected on the kanamycin-containing TGY agar, and the modified chromosomal region was PCR-amplified and sequenced to confirm the gene replacement.

The pinene production strains were generated by transformation of *D. radiodurans* strains R1, ∆*dr0862*, and ∆*dr1395* with relevant expression plasmids. All plasmids and strains are listed in Table 1.

**Table 1:** Plasmids and strains

Name	Description	Source
*Plasmids*		
pRADN1	*E. coli-D. radiodurans* shuttle vector, chloramphenicol resistance	Hirofumi Ohba et al., 2005
pET-26b	*E. coli* expression vector, kanamycin resistance	Novagen
pMBIS	*E. coli* mevalonate pathway vector	Martin et al., 2003
pET26b-*ps*_*Dr*_	pET-26b-*ps*_*Dr*_	This study
pRAD-kP	pRADN1-P_*katA*_-*ps*_*Dr*_	This study
pRAD-gP	pRADN1-P_*groE*_-*ps*_*Dr*_	This study
pRAD-tP	pRADN1-P_*tufA*_-*ps*_*Dr*_	This study
pRAD-P-I	pRADN1-P_*katA*_-*ps*_*Dr*_-P_*katA*_-*ispA*	This study
pRAD-P-I-D	pRADN1-P_*katA*_-*ps*_*Dr*_ -P_*katA*_-*ispA*-P_*katA*_-*dxs*	This study
pRAD-P-D	pRADN1-P_*katA*_-*ps*_*Dr*_ -P_*katA*_-*dxs*	This study
pRAD-I	pRADN1-P_*katA*_-*ispA*	This study
pRAD-P:G-D	pRADN1-P_*katA*_- *ps*_*Dr*_:*ispA*-P_*katA*_-*dxs*	This study
pRAD-G:P-D	pRADN1-P_*katA*_-*ispA*:*ps*_*Dr*_-P_*katA*_-*dxs*	This study
pET-26b- *dr0862*::kan^R^	pET-26b-*dr0862*::kan^R^	This study
pET-26b- *dr1395*::kan^R^	pET-26b-*dr1395*::kan^R^	This study
*Strains*		
*E. coli* BL21(DE3)	F ֿ ompT hsdSB (rB ֿ mB ֿ) gal dcm rne131 λ(DE3)	Invitrogen
*E. coli* NM522	*sup*E, *thi*, Δ(*lac-pro*AB), *hsd*5 (r ֿ, m ֿ)	Promega
*D. radiodurans* R1	wild type	ATCC 13,939
WT kP	*D. radiodurans* pRAD-kP	This study
WT gP	*D. radiodurans* pRAD-gP	This study
WT tP	*D. radiodurans* pRAD-tP	This study
WT P-I	*D. radiodurans* pRAD-P-I	This study
WT P-I-D	*D. radiodurans* pRAD-P-I-D	This study
∆*dr1395* I	*D. radiodurans* ∆*dr1395* pRAD-I	This studys
∆*dr1395* P-I	*D. radiodurans* ∆*dr1395* pRAD-P-I	This study
∆*dr1395* P-I-D	*D. radiodurans* ∆*dr1395* pRAD-P-I-D	This study
∆*dr0862*	*D. radiodurans* ∆*dr0862*	This study
∆*dr0862* kP	*D. radiodurans* ∆*dr0862* pRAD-P	This study
∆*dr0862* P-I	*D. radiodurans* ∆*dr0862* pRAD-P-I	This study
∆*dr0862* P-D	*D. radiodurans* ∆*dr0862* pRAD-P-D	This study
∆*dr0862* P-I-D	*D. radiodurans* ∆*dr0862* pRAD-P-I-D	This study
∆*dr0862* P:G-D	*D. radiodurans* ∆*dr0862* pRAD-P:G-D	This study
∆*dr0862* G:P-D	*D. radiodurans* ∆*dr0862* pRAD-G:P-D	This study
*E. coli* EcpsB	*E. coli* BL21 pMBIS pET26b-*ps*_*Dr*_	This study

To benchmark the constructed *D. radiodurans* strains against an *E. coli* pinene production strain, *E. coli* EcpsB strain was constructed. To do this, we subcloned the *ps*_*Dr*_ gene and inserted it in the pET26-b expression vector between *Nde*I and *Bam*HI to generate pET26b- *ps*_*Dr*_. Next, *E. coli* BL21(DE3) was co-transformed with pET26b- *ps*_*Dr*_ and the pMBIS plasmid encoding the mevalonate pathway from *Saccharomyces cerevisiae* [27]. 1 mM IPTG added for gene expression induction and pinene production in *E. coli* EcpsB. Whereas the *ps*_*Dr*_ gene was codon optimized for expression in *D. radiodurans*, production of the enzyme in *E. coli* was confirmed by SDS-PAGE (Additional file 1: Fig. S5).

#### Deinococcus radiodurans transformation

The transformation was performed using a modified calcium chloride method [28]. Briefly, *D. radiodurans* was grown in PGY at 30 °C and agitation speed of 180 rpm for 24 h. 100 µl of the bacterial culture was added to a new 5 mL of PGY and grown at 30 °C and 180 rpm shaking to reach OD_600_ of 0.4. 1.5 mL of the cell culture was centrifugated, and the supernatant was discarded. The cell pellet was resuspended in 100 µL of the liquid PGY plus 40 µL of 0.3 M calcium chloride and aliquoted in 30 µL microtubes. 10 µl of the target plasmid (1–2 µg) or purified amplicon (0.2–0.6 µg) was added to the microtubes and mixed by gentle pipetting. This mixture was placed on ice for 15 min and then placed in a 30 °C incubator for 2 h. Subsequently, 1 mL PGY was added to the bacteria, and the mix was further incubated at 30 °C for 24 h. After this incubation, the mix was plated on TGY agar plates containing chloramphenicol/kanamycin (or both) and was incubated at 30 °C for 72 h.

#### Confirmation of gene expression from groE, katA and tufA promoters

The Real-time PCR method was performed to confirm that the promoters were functional, leading to transcription of the pinene synthase gene. RNA extraction, cDNA synthesis, and SYBR green qPCR were performed with the corresponding kits according to the manufacturer’s instructions (Pars Toos Co.). The primer sets Ps-RT fwd/rev and Gap-RT fwd/rev were used for the amplification of pinene synthase gene and the housekeeping gene (glyceraldehyde 3-phosphate dehydrogenase (*dr1343*)), respectively.

Moreover, to confirm the presence of the pinene synthase in the cell, we grew *D. radiodurans* expressing pinene synthase fused to GFP, for 24 h in PGY medium at 30 °C shaking at 180 rpm. The expression of pinene synthase-GFP fusion was visualized using fluorescence microscopy (Leica DM, 4000 B).

#### Pinene toxicity assay

To investigate the toxicity of α- and β-pinene, engineered pinene producing strains *D. radiodurans* (∆*dr0862* P-I-D strain) and *E. coli* EcpsB were cultivated in the presence of exogenous pinene in a microtiter plate. These strains were cultivated in the rich medium broth at 30 °C and 180 rpm agitation speed for 24 h and inoculated the rich medium to a starting OD_630_ of 0.02. Different concentrations of α- and β-pinene (1, 1.75, 2.5, and 5 g/L) were added to the medium and incubated at 30 °C with shaking speed set to “medium” in a microplate reader (BioTek, ELx808). The growth of different strains was determined by measuring OD_630_ every 30 min for 48 h.

#### Testing the ability of D. radiodurans to metabolize pinene

To determine the ability of *D. radiodurans* to metabolize α- and β-pinene, the rich medium with 10% v/v dodecane was supplemented with 20 mg/L α- and β-pinene*. D. radiodurans* was inoculated to OD_600_ 0.02 and cultured for 5 days at 30 °C and 180 rpm shaking. Thereafter, 0.5 mL of the dodecane layer was transferred to a 1.5 ml microcentrifuge tube and centrifuged at 14,000 g for 5 min to precipitate debris. The upper layer was placed in a new microtube, and this sample was analyzed by GC-FID as described below. The obtained results were compared with those of bacterium-free and the dodecane layer with added pinene isomers before fermentation.

#### Pinene sample preparation

We assessed the pinene extraction in various dodecane concentrations (10 and 20%) in the rich and minimal glycerol medium. Regarding the fermentation for the pinene production, 1 mL of a 24-h old bacterial culture was added to 50 mL of fresh growth medium and incubated at 30 °C and 180 rpm shaking for the rich and 100 rpm for minimal glycerol medium. When the OD_600_ reached 3 for *D. radiodurans* and 1 for *E. coli*, 10% *v/v* dodecane for the rich medium (180 rpm shaking) and 20% for minimal glycerol medium (100 rpm shaking) was added to extract pinene. The dodecane layer was sampled after 24, 48, and 72 h, and the pinene recovered therein was measured through GC [29].

### GC-FID and GC–MS analysis

Pinene production by *D. radiodurans* and *E. coli* was measured by gas chromatography (GC). To this end,, 0.5 mL of the dodecane layer from a shake flask culture was transferred to a 1.5 ml microcentrifuge tube and was then centrifuged at 14,000 g for 5 min to precipitate debris. The upper layer was placed in a new microtube, and 1 µl of that was injected into GC-FID or GC–MS. Moreover, to analyze the intracellular presence of produced pinene, the biomass of pinene production fermentation was separated by centrifugation in 10.000 g for 10 min, and 5 ml dodecane was added to the biomass. After vortexing vigorously, the cells were lysed by sonication (20 s sonication/5 s rest for 15 min on ice). Afterwards, 0.5 mL of the dodecane was transferred to a 1.5 ml microcentrifuge tube and was centrifuged at 14,000 g for 5 min to precipitate debris. The upper layer dodecane was transferred to a new microtube and was analyzed by GC-FID.

For optimization and finding the retention time, α- and β-pinene molecules (Sigma Co.) were used as the standard samples. In addition, to quantify the production rate, various pinene concentrations were injected into the GC-FID device to plot the standard curve.

The GC-FID (Thermo Scientific Trace 1300) was used with a BPX-5 column (30 m 0.22 mm × 0.25 µm). The inlet temperature was set to 300 °C, purge flow 5 ml/min, split flow at 180 ml/min, the oven at 50 °C for 1 min, ramp at 50 °C/min to 250 °C.

The produced pinene molecule, by engineered strains, was confirmed by GC–MS. For the GC–MS, samples were analyzed by a gas chromatography system (Thermo Scientific Trace 1300) coupled to a single quadrupole mass spectrometer (Thermo Scientific ISQ 7000). Split injection was used, with a ratio of 20:1 with an inlet temperature of 300 °C. The carrier gas was helium, and the inlet flow was set at 2 mL·min^−1^ throughout the run. The gas chromatography system was equipped with a Thermo Scientific TraceGOLD TG-5MS column (length: 30 m; diameter: 0.25 mm; film thickness: 0.25 µm), and the following temperature gradient was utilized: Initial temperature of 40 °C held for 10 min, raised to 250 °C at 50 °C min^−1^, and then held at 250 °C for 8 min. Electron impact ionization was used with the mass detector set to 30–600 m/z. The transfer line was held at a constant 280 °C, and the ion source temperature was 300 °C.

## Results and discussion

### Potential of *D. radiodurans* as a pinene production host

In the present study, we investigated the potential of using *D. radiodurans* as a novel host for the production of the monoterpene molecule pinene using glycerol as a substrate. We hypothesized that *D. radiodurans* could be particularly well suited due to the fact that it produces a pigment, deinoxanthin, that like pinene is synthesized from GPP. Further, *D. radiodurans* shows resistance to many types of stress, and we considered the possibility that it would have a higher inherent resistance to pinene and GPP than, for example *E. coli*. Additionally, *D. radiodurans* has relatively high intracellular concentration of manganese (0.2–4 mM), which is the main cofactor for the pinene synthase enzyme [22].

**Fig. 1:**
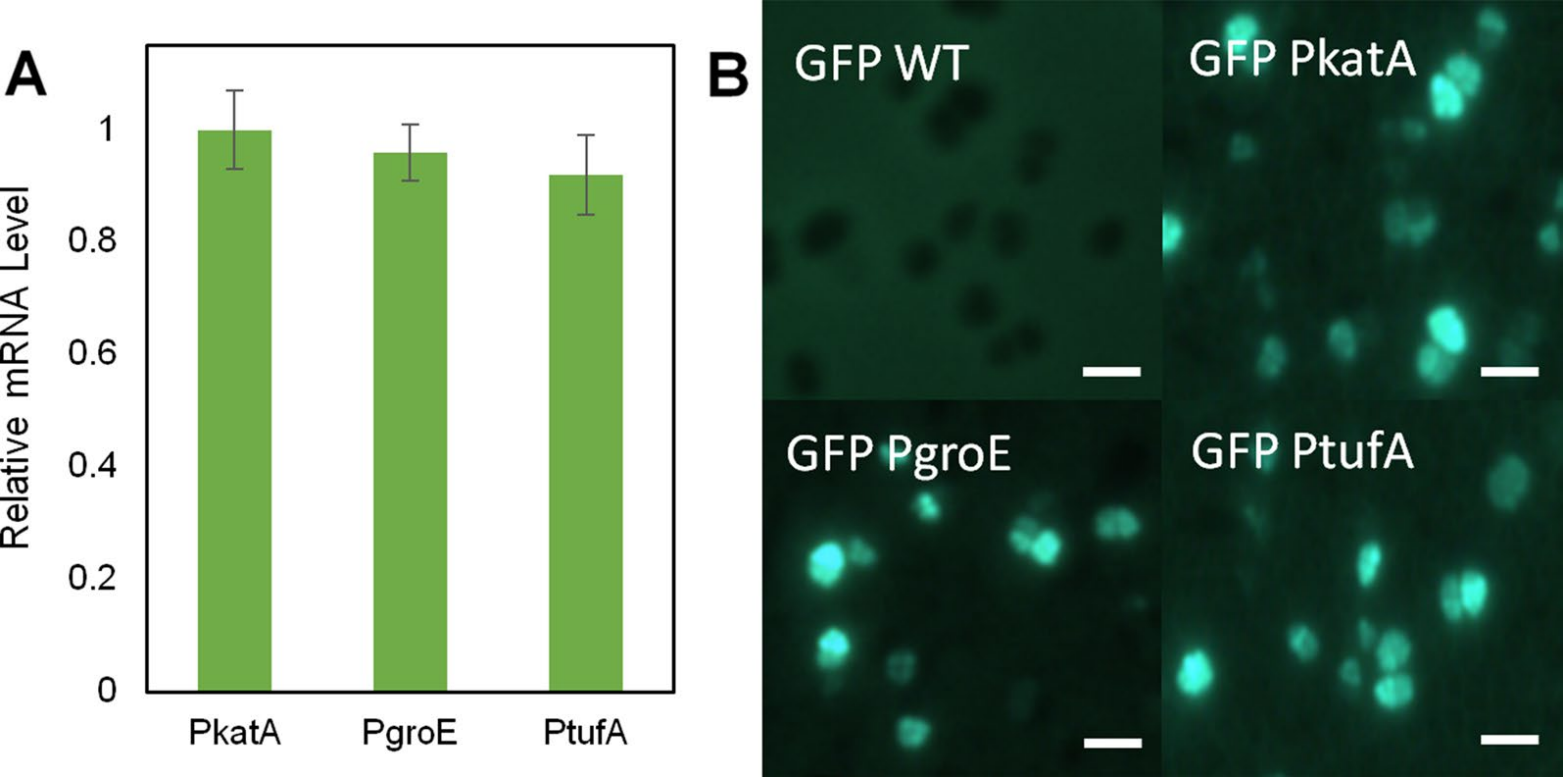
Pinene synthase-encoding gene is expressed in wild type *D. radiodurans* cells. **a** The expression levels from three different promoters P_*katA*,_ P_*groE*,_ and P_*tufA*_ were analysed by real-time PCR, **b** and the presence of pinene synthase was confirmed using a construct expressing the pinene synthase gene fused to *gfp*. Fluorescence in the cells was observed by fluorescence microscopy. Scale bars: 4 µm

As a first step, we attempted to construct a basic pinene production strain. The precursor for pinene is GPP. This molecule is produced via the native non-mevalonate pathway, where it acts as a precursor for the pigment molecule deinoxanthin. Conversion of GPP to pinene is catalyzed by pinene synthase. Several pinene synthases have previously been described, and we selected the pinene synthase from *A. grandis* that was shown to be functional in *E. coli* [16]. The coding sequence was codon-optimized for *D. radiodurans* and placed under the control of the three promoters P_*katA*,_ P_*groE*,_ and P_*tufA*_ that have been employed previously for gene expression in *D. radiodurans* [26, 30]. We then cultured these strains (WT kP, WT gP, and WT tP) for 72 h in the rich medium containing glycerol, but pinene was not detected in the fermentation broth. To assure that the genes were expressed, we performed real-time PCR. This demonstrated that the genes were in fact transcribed, but also that the mRNA levels were similar for all three promoters (Fig. 1a). The use of the promoter P_*katA*_ has been reported previously [26, 28]. Considering that the difference between that and the other promoters tested was minimal, we decided to use P_*katA*_ for all subsequent genetic constructs. To assure that the pinene synthase protein was also present in the cell, we expressed a pinene synthase variant tagged with GFP in the C-terminus. Our fluorescence microscopy analysis confirmed that the protein was indeed produced in the cells (Fig. 1b).

### Metabolic engineering strategy for pinene production in *D. radiodurans*

**Fig. 2:**
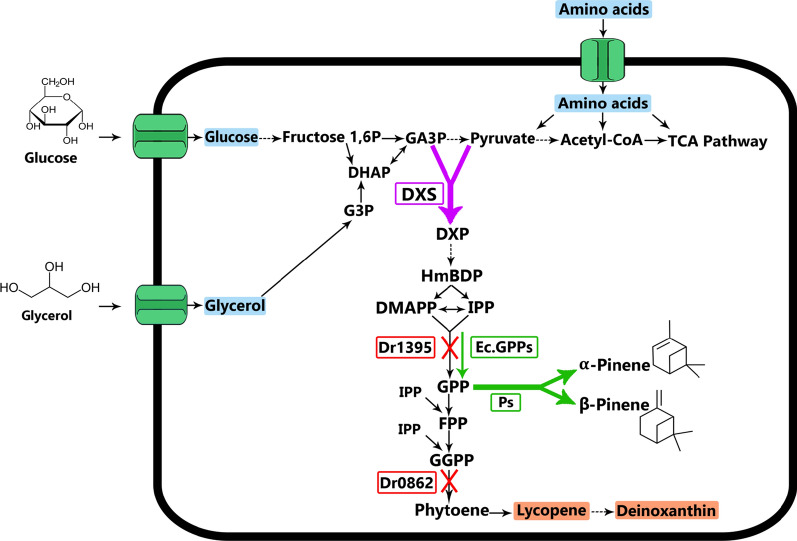
Schematic representation of the pinene production strategy and modifications to enhance the production in *D. radiodurans*. *D. radiodurans* carbohydrate sources including glycerol, glucose, and amino acids catabolism and pyruvate (Pyr) production in the cell. Pyr with glyceraldehyde 3-phosphate (GA3P) is converted to 1-Deoxy-D-Xylulose 5-Phosphate (DXP) by the enzyme 1-deoxy-d-xylulose-5-phosphate synthase (DXS). Following this pathway, isopentenyl diphosphate (IPP) and dimethylallyl diphosphate (DMAPP) molecules are produced. Afterward, GPP synthase produces a GPP molecule from these two molecules. Finally, pinene synthase (Ps) catalyzes synthesis of pinene by cyclizing GPP. Black arrows indicate the natural path inside the cell; purple arrows indicate overexpression in the pathway; green arrows indicate heterologous expression, and the cross sign indicates the inactivation of the gene in the cell. The abbreviations of the pathway are as follows: Ec. GPPs, *E. coli* GPP synthase gene; Dr1395, native GPP synthase; Dr0862, native phytoene synthase

Our initial efforts demonstrated that the successful expression of pinene synthase is not sufficient to produce detectable levels of pinene. Since we had no reason to doubt that the expressed pinene synthase is functional, we considered different possible explanations based on the known metabolic pathways in *D. radiodurans* (Fig. 2). It is evident that the native GPP synthase plays an important role in the process by catalyzing the synthesis of GPP. While *D. radiodurans* is known to produce sufficient amounts of GPP for deinoxanthin production, there is a possibility that the GPP pool is drained by the subsequent reactions in the deinoxanthin pathway: condensation of GPP units to produce farnesyl pyrophosphate (FPP) and geranylgeranyl pyrophosphate (GGPP). These reactions are consuming GPP and are therefore competing with pinene synthesis for the substrate GPP.

Liu and coworkers previously indicated thatthe GPP synthase is essential in *D. radiodurans*, but could be inactivated when complemented with *E. coli* GPP synthase IspA that does not catalyze GGPP synthesis [31].Their results showed GPP and FPP, as well as GGPP, are the main products of the native GPP synthase enzyme in *D. radioduran*, and the native GPP synthase does not only produce GGPP. Therefore, such a strain without producing GGPP should be beneficial for pinene production, as it preserves the GPP pool and high amount of carbon flux by abolishing deinoxanthin synthesis. An alternative way to prevent deinoxanthin production without deleting of the native GPP synthase in *D.* radiodurans would be via the inactivation of phytoene synthase that catalyzes the conversion of GGPP to phytoene. In case the synthesis of GPP from DMAPP and IPP would be a bottleneck, expression of the GPP synthase IspA should also lead to an increase in the GPP pool. Finally, Previous studies have shown that using heterologous mevalonate pathway could enhance the GPP pool and terepene production in *E. coli* [15], but studies on non-mevalonate pathway in *D. radiodurans* showed this pathway is highly active, and we decided to boost this native pathway.One could consider improving the flux through the entire pathway by overexpressing DXS to direct more GA3P and pyruvate through the pathway via DXP synthesis. Based on this analysis, we decided to inactivate the genes encoding GPP synthase and phytoene synthase and to make plasmids for the expression of pinene synthase and GPP synthase IspA and/or DXS.

### Evaluation of engineered* D. radiodurans* strains for pinene production

**Fig. 3:**
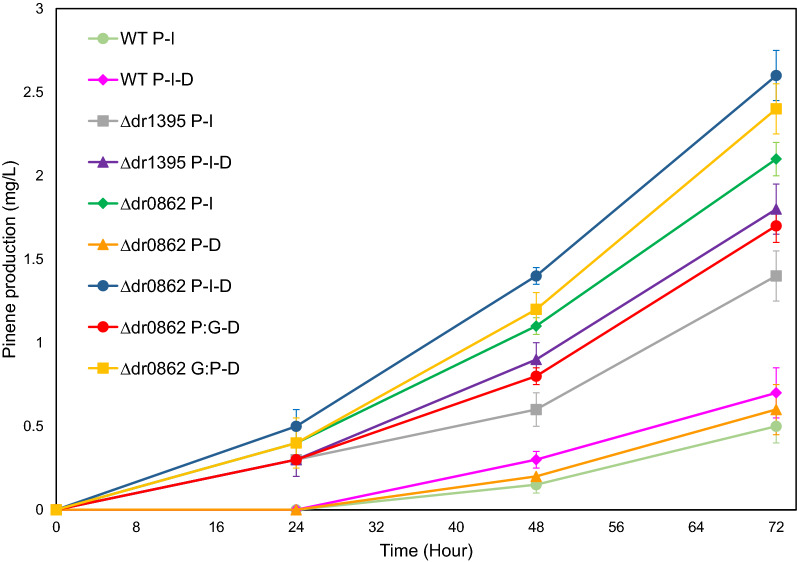
The amounts of pinene produced by different recombinant *D. radiodurans* strains during 72 h of growth in the rich medium supplemented with glycerol, in shake flasks

Based on our analysis of the metabolism of *D. radiodurans*, we constructed a number of strains that, besides the expression of pinene synthase, expressed *E. coli* GPP synthase and/or overexpressed DXS. This was done in wild type as well as in strains where either the native GPP synthase (∆*dr1395*) or the phytoene synthase (∆*dr0862*) was inactivated. These strains were subsequently tested for pinene production in the rich medium by GC-FID, and pinene isomers confirmed in the final samples by GC–MS (Table 2 and Fig. 3).

**Table 2:** Pinene production in *D. radiodurans* after 72 h of shake flask fermentation

Host	Plasmid-expressed proteins	Titer [mg/L]	Productivity [ug pinene/h]	Yield [mg pinene/g glycerol]	Medium
WT	PS-IspA	0.5 ± 0.1	7	–	Rich Medium
	PS-IspA-DXS	0.7 ± 0.1	10	–	Rich Medium
∆*dr1395*	PS-IspA	1.4 ± 0.2	19	–	Rich Medium
	PS-IspA-DXS	1.8 ± 0.2	25	–	Rich Medium
∆*dr0862*	PS-IspA	2.1 ± 0.1	29	–	Rich Medium
	PS-DXS	0.6 ± 0.1	8	–	Rich Medium
	PS:IspA-DXS	1.7 ± 0.1	24	–	Rich Medium
	IspA:PS-DXS	2.4 ± 0.2	33	–	Rich Medium
	PS-IspA-DXS	2.6 ± 0.2	36	–	Rich Medium
	PS-IspA-DXS	3.2 ± 0.2	45	0.13	Minimal Glycerol Medium
*E. coli* EcpsB	MEV-PS	0.9 ± 0.2	13	–	Rich Medium
*E. coli* EcpsB	MEV-PS	0.65 ± 0.1	9	0.026	Minimal Glycerol Medium

As mentioned above, expression of *A. grandis* pinene synthase in the wild type strain (WT kP) did not lead to the production of detectable levels of pinene. When *E. coli* GPP synthase was introduced (WT P-I), we detected production of 0.5 mg/L of pinene. This would seem to indicate that the GPP pool was not sufficient in the strain (WT kP), and that conversion of DMAPP and IPP to GPP indeed presented a bottleneck for pinene production.

We then overexpressed DXS in this strain, resulting in strain WT P-I-D, which was expected to increase the production of 1-deoxy-d-xylulose-5- phosphate (DXP) from pyruvate (Pyr) and 3-glyceraldehyde phosphate (GA3P), and thereby increase the flux through the non-mevalonate pathway. It has previously been shown that among the enzymes of this pathway, only overexpression of this enzyme can increase the production of GPP and carotenoids in *D. radiodurans* significantly [32], but in our experiments, it did not lead to a significantly higher pinene titer.

Previous studies have shown that the non-mevalonate pathway in *D. radiodurans* can support the production of 203.5 mg/g dried cell weight of carotenoid molecules in fed-batch fermentation [32]. These results indicate a high flow rate of GPP into the carotenoid pathway that leads to deinoxanthin production. Consequently, it should be beneficial with respect to pinene synthesis to disrupt this pathway. To test this, we constructed two strains where genes encoding the native GPP synthase (*dr1395*) and the phytoene synthase (*dr0862*), respectively, were inactivated. As mentioned above, the *dr1395* mutant could only be constructed when complemented with the *E. coli* GPP synthase. By expressing the pinene synthase, this strain (∆*dr1395* P-I) enabled the production of 1.8 mg/L of pinene, which was a 3.6 fold improvement compared to the titer obtained in the wild type background (WT P-I). When further overexpressing the DXS enzyme, in strain ∆*dr1395* P-I-D we recorded a slightly higher titer, but the difference was not statistically significant. As mentioned above, the substitution of *D. radiodurans* GPP synthase with that of *E. coli* prevents the formation of GGPP, which feeds into the deinoxanthin biosynthesis pathway. It thus prevents draining the GPP pool via deinoxanthin synthesis, but also prevents the possible buildup of GGPP. On the other hand, it might reduce the amount of GPP.

To perform a more direct inactivation of the deinoxanthin pathway, we inactivated the gene *dr0862*, encoding phytoene synthase. When evaluating pinene production of this strain expressing pinene synthase and *E. coli* GPP synthase (∆*dr0862* P-I), we saw a further increase in titer to 2.1 mg/L, which was a 1.5-fold increase compared to that observed in the *dr1395* mutant (∆*dr1395* P-I). This would suggest that the primary beneficial effect of the mutations is the prevention of deinoxanthin formation and not preventing the conversion of GPP to FPP and GGPP catalyzed by the *D. radiodurans* GPP synthase per se. In this strain, the additional expression of DXS (∆*dr0862* P-I-D) to increase flux through the non-mevalonate pathway led to a small but significant increase in pinene titer (2.6 mg/L).

Previous reports have shown that expression of fusion forms of pinene synthase and GPP synthase resulted in increased pinene production in *E. coli*, with the fusion form containing pinene synthase in the C-terminus increasing the titer by sixfold [16]. We constructed versions of fusion proteins containing *A. grandis* pinene synthase (in either N- or C-terminus) and *E. coli* GPP synthase. Then we tested the performance of the fusion proteins in the *dr0862* mutant overexpressing DXS (∆*dr0862* P:I-D and ∆*dr0862* I:P-D). Both fusion proteins were active (at least with respect to pinene synthase activity). As in *E. coli*, the variant containing C-terminal pinene synthase (∆*dr0862* I:P-D) performed best, but it did not lead to a further improvement in pinene titer.

**Fig. 4:**
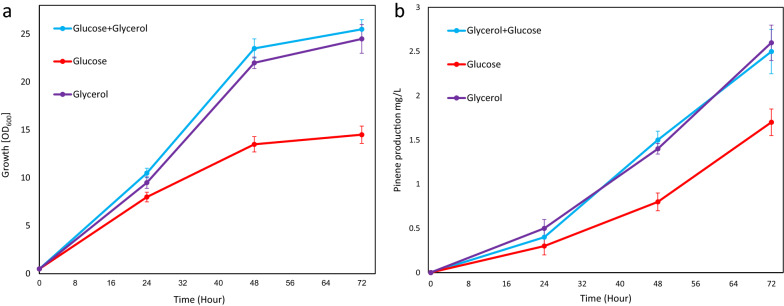
**a** Growth and **b** the pinene production of *D. radiodurans* in glucose- and glycerol-supplemented media. *D. radiodurans* was grown in a rich medium supplemented with 5 g glucose, 5 g glycerol, 2.5 g of each for a period of 72 h in shake flasks at 30 °C. All the experiments were done in biological triplicates

For initial evaluation of the engineered strains, we used a rich medium supplemented with glycerol. To rule out the possibility that glycerol could be an inferior substrate, we compared the growth and pinene production in various media where glycerol was substituted with either glucose or a mix of glycerol and glucose (Fig. 4). This demonstrated that glycerol in the medium supported a higher pinene titer than did glucose.

### Pinene resistance and degradation in *D. radiodurans*

Previous investigations have revealed that pinene is toxic for fungi and bacteria. Pinene minimal microbicidal concentrations for various species such as *Cryptococcus neoformans* (0.12 g/L), *Candida albicans* (3.12 g/L), methicillin-resistant *Staphylococcus aureus* (4.15 g/L), and *Corynebacterium glutamicum* (< 2.5 g/L) are reported, which show high toxicity of this monoterpene on microorganisms [29, 33, 34]. On the other hand, pinene toxicity has been reported as one of the main problems for producing pinene by microbial cell factories [16]. Also, enhancing the pinene resistance by overexpression of efflux pumps in *E. coli* and mutagenesis in the strain caused improvement in pinene production by *E. coli* [17].

**Fig. 5:**
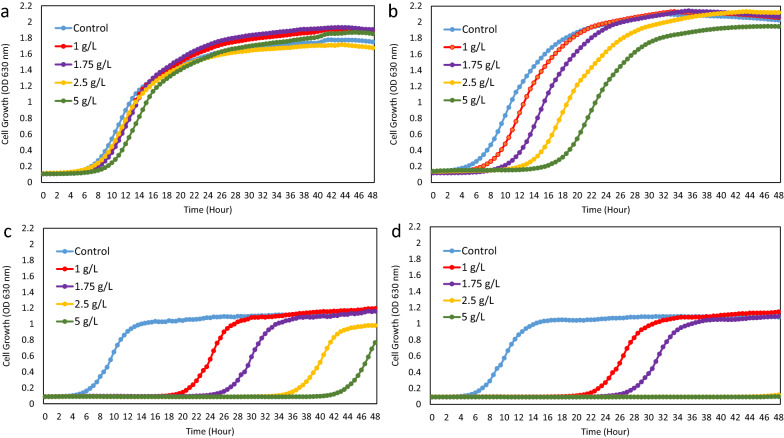
Pinene resistance of *D. radiodurans ∆dr*0862 P-I-D and *E. coli* EcpsB strains. The growth of the strains in the rich medium containing various concentrations of **a** α-pinene (*∆dr0862* PID strain), **b** β-pinene (*∆dr0862* PID strain), **c** α-pinene (*E. coli* EcpsB strain), and **d** β-pinene (*E. coli* EcpsB strain) was followed in microtiter plates for 48 h. The growth curves at different pinene concentrations are shown. The experiment was done with three biological replicates

Considering the possibility that pinene production would be affected due to its toxic effect, we found it relevant to investigate the pinene tolerance in the *D. radiodurans* ∆*dr0862* P-I-D strain. This was done by adding exogenous pinene isomers to the medium in concentrations between 1 and 5 g/L of pinene isomers. We also assessed *E. coli* EcpsB strain, grown in the same medium, in order to compare the strains in terms of pinene resistance. Growth was followed for 48 h, and the most obvious effect of pinene was an extended lag-phase for the cells. *D. radiodurans* showed high resistance to α-pinene, and growth was only mildly affected at the highest tested concentration (5 g/L). In contrast, the *E. coli* EcpsB strain was strongly affected even at the lowest concentration used (1 g/L) with a 14 h longer lag phase. For both strains, β-pinene was more toxic. In case of *D. radiodurans*, at 1 g/L a slight increase in the lag phase, which was exacerbated to a 12 h increase in the lag phase at 5 g/L. For *E. coli*, β-pinene increased the lag phase (slightly more than α-pinene) at the lower concentrations, and completely inhibited growth at a concentration of 2.5 g/L (Fig. 5).

The results however revealed that *D. radiodurans* possesses a relatively high tolerance to α- and β-pinene. It is evident from the tolerance data that the amount of the pinene produced by *D. radiodurans* ∆*dr0862* P-I-D strain is well below the inhibitory concentration and thus would be unlikely to negatively affect the pinene production. Also, our *E. coli* pinene production strain, exhibited a higher sensitivity to pinene than *D. radiodurans*. In a previous study, Sarria et al*.* reported that *E. coli* shows resistance to α-pinene concentrations below 4.3 g/L, and that β-pinene (up to 10 g/L) showed no effect on its growth. Conversely, our results indicated that both α- and β-pinene inhibit the growth of *E. coli* EcpsB. The reason for this discrepancy is presently not known but could perhaps reside in differences in growth conditions.

Monoterpene molecules have different effects on cells, which can cause toxicity impacts. Among them, inhibition of enzymes activity, preventing biofilm formation, reducing mitochondrial activity in mammalian cells [33], and membrane fluidization changes [35] are well studied. Based on the knownmechanisms of monoterpene toxicity on different cells, various protein protection and antistress mechanisms in *D. radiodurans* plus the particular multilayered structure of its membrane and cell wall could be the reason for the observed higher tolerance [22].

To rule out the possibility that pinene was metabolized in the cell, we conducted long-term fermentation experiments (120 h) where α- and β-pinene were added to the *D. radiodurans* growth medium. As the amount of pinene measured throughout the entire experiment was almost identical to that of the control sample, not inoculated with *D. radiodurans*, we concluded that pinene is not metabolized (Additional file 1: Fig. S2).

Moreover, to study the possibility of pinene accumulating in the cells, we resuspended the biomass after fermentation in dodecane and disrupted the cells by sonication. GC-FID analysis of the samples did not allow detection of pinene, suggesting that most or all of the produced pinene was transferred to the extracellular medium.

### Pinene production in minimal glycerol medium

**Fig. 6:**
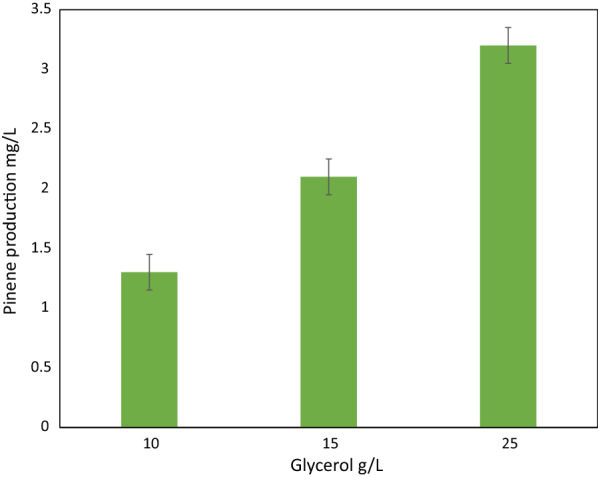
Pinene production in different concentrations of glycerol in minimal glycerol medium by *D. radiodurans* ∆*dr0862* P-I-D

Next, we wanted to assess whether pinene could also be produced in a minimal medium with glycerol as the primary carbon source. To this end, we made a minimal medium consisting 25 g/L of glycerol devoid of the complex carbon sources peptone and yeast extract. In this medium, the best pinene producing strain *D. radiodurans* ∆*dr0862* P-I-D produced 3.2 mg/L of pinene with a yield of 0.13 ± 0.04 mg/g glycerol and productivity of 45 µg pinene/h in 72 h in shake flasks (Table 2). Moreover, we tested different concentrations of glycerol (10, 15, and 25 g/L) in the minimal medium to evaluate pinene production dependency on glycerol concentration in the medium (Fig. 6).

In this case, we observed that lower agitation speed and a higher percentage of dodecane (20% instead of 10%), was necessary for optimal pinene production and extraction in the fermentation (Additional file 1: Fig. S3). Using glycerol as the main source of carbon for fermentation has been an attractive venue for years, and the production of different molecules such as 1-propanol [7], γ-terpinene [36], limonene [37], and various enzymes [38] from glycerol using cell factories have been reported. With respect to *D. radiodurans*, this is, to the best of our knowledge, the first report describing the use of a glycerol-based minimal medium (without protein source and yeast extract), and our results show a potential for growth and production of terpene molecules from glycerol using this bacterium.

### Pinene production in *D. radiodurans* and *E. coli*

In this study, we are reporting the maximal production of 3.2 mg/l of pinene in a minimal glycerol medium in shake flask cultures by *D. radiodurans*. In previous studies on pinene production, Kang et al*.* [29] could construct a *C. glutamicum* strain by the expression of GPP synthase,pinene synthase from *A. grandis* plus overexpression of *dxs* and isopentenyl diphosphate isomerase. This engineered *C. glutamicum* strain could produce 0.177 mg/L pinene. In a recent report for pinene production, Xiaomin Wu et al*.* [39] engineered a *Rhodobacter sphaeroides* strain to produce pinene. Expression of a fusion protein of GPP synthase and pinene synthase in *R. sphaeroides* led to 0.098 mg/L pinene, and more modifications yielded a production of 0.5 mg/L in this strain. These pinene production titers indicate that *D. radiodurans* could be an appropriate host for pinene production. The titer obtained in this study, however, was lower than the highest titers obtained in a highly engineered *E. coli* strain (166.5 mg/L). This pinene production titer by *E. coli* strain was achieved by many manipulationsand mutagenesis (Table 3). In order to address the question of whether *D. radiodurans* is inherently a better host for pinene production than *E.coli*, we constructed a more basic *E. coli* production strain, comparable to the genetic makeup of our best *D. radiodurans* production strain (∆*dr0862* P-I-D), that express only the pinene synthase and the mevalonate pathway of *S. cerevisiae*. This *E. coli* strain was evaluated for pinene production under the same conditions as for the *D. radiodurans* strains. The *E. coli* strain produced 0.9 mg/L and 0.65 mg/L pinene, in the rich and the minimal glycerol medium, respectively. These amounts were 2.9 and 4.9 fold lower than the highest titer observed in *D. radiodurans*. While this is of course an artificial comparison, it does support the notion that *D. radiodurans* could represent a useful host organism for the production of monoterpenes, such as pinene (Table 2).

**Table 3:** Microbial engineered strains for pinene production

Host	Engineering design	Production mg/L	References
*E.coli*	Heterologous mevalonate pathway expression, *IspA* overexpression, pinene synthase from *P. taeda*	5.44 (Shake Flask) 970 (Fed-batch)	[15]
*E. coli*	GPP synthase and pinene synthase fusion protein from *A.grandis* expression*,* heterologous mevalonate pathway expression	32	[16]
*E. coli*	Heterologous mevalonate pathway expression, *idi* and GPP synthase overexpression, mutagenesis of Pinene synthase from *P. taeda*	140	[40]
*E. coli*	Pinene synthase enzyme modifications, modular co-culture system engineering to modularize the heterologous mevalonate pathway expression, increasing the pinene tolerance by overexpression of the efflux pumps and mutagenesis by adaptive laboratory evolution after atmospheric and room temperature plasma (ARTP)	166.5	[17]
*C. glutamicum*	Expression of GPP synthase from *P. taeda* and pinene synthase from *A. grandis* plus overexpression of *dxs* and *idi*	0.177	[29]
*R. sphaeroides*	Expression of fusion protein of GPP synthase and pinene synthase from *A.grandis* (0.098 mg/L pinene), and ribosomal binding site optimization, overexpressing of *dxs*, 1-deoxy-d-xylulose 5-phosphate reductoisomerase, and *idi* (0.5 mg/L pinene)	0.5	[39]

#### D. radiodurans produces exclusively the β-pinene isomer

When the *A. grandis* pinene synthase was expressed in *E. coli*, it led to the production of mix of pinene isomers (42% α- and 58% β-pinene) [16]. In contrast, when we expressed this pinene synthase in *D. radiodurans,* we observed the formation of the β-pinene exclusively (Additional file 1: Fig. S4). It has previously been reported that a fusion of pinene synthase with *E. coli* GPP synthase allows production of the two isomers in a 50:50 ratio in *E. coli* [16]. Also, for this fusion protein (∆*dr0862* I:P-D), we observed exclusively β-pinene production in *D. radiodurans*. This could indicate that the intracellular conditions of *D. radiodurans* might alter the behavior of pinene synthase, resulting in the exclusive production of β-pinene.

## Perspectives

In this study, we demonstrated that *D. radiodurans* can be engineered to produce pinene. The high resistance of this bacterium to toxic molecules, various sources of stress, and its ability to grow on various carbon sources could make *D. radiodurans* an appropriate candidate for the production of pinene. Such production setup would result in the production of a valuable second-generation biofuel by utilization of lignocellulosic compounds and nutritional municipal/industrial wastes. However, for this to be economically feasible, it will be necessary to further develop the *D. radiodurans* pinene production strain. Possible strategies to do this could include evaluating alternative GPP synthases or its mutants to prevent the FPP side reaction, using mutagenesis techniques, pinene transportation modifications, and fine-tuning protein levels and redox balance in the cell. Further, harboring an optimized mevalonate pathway, enhancement of upstream pathways, and the potential of optimizing growth conditions during production should be evaluated. It has been shown that the use of high cell-density cultures for carotenoid production in *D. radiodurans* could increase the production of terpene molecules significantly [21, 32], and this approach can be used for pinene production as well.

## Conclusions

To the best of our knowledge, this study is the first report on monoterpene production in *D. radiodurans* using a minimal glycerol medium. By inactivating phytoene synthase and expressing the *A. grandis* pinene synthase, the *E. coli* GPP synthase, and overexpressing DXS, 3.2 mg/L pinene with a yield of 0.13 mg pinene/g glycerol and productivity of 45 µg pinene/h was achieved in a minimal medium in shake flask culture. In a comparable setup, the *E. coli* EcpsB strain produced 0.65 mg/L pinene with a yield of 0.026 mg pinene/g glycerol. Our results demonstrate a potential of using *D. radiodurans* as a cell factory for pinene production.

## Supplementary Information

**Additional file 1: Table S1**. List of oligos used for vector construction and real-time PCR. **Figure S1**. The sequence of the pinene synthase-encoding gene codon-optimized for *D. radiodurans.***Figure S2**. Pinene degradation or consumption by *D. radiodurans* in 120 h. *D. radiodurans* was grown in a medium supplemented with 20 mg/L of pinene isomers and residual pinene was quantified by GC-FID after 120 h. Un-inoculated pinene supplemented medium was used as the control. **Figure S3**. Effect of using different concentrations of dodecane (10% and 20%) **a** in the rich and **b** the minimal glycerol medium and various agitation speed (70, 100, 180, and 250 rpm) on the pinene production by *D. radiodurans* ∆*dr0862* P-I-D*.* **Figure S4.** GC-FID chromatograms. **a** Alpha-pinene standard, **b** beta-pinene standard, **c** beta-pinene production by *D. radiodurans*, and **d** alpha- and beta-pinene production by *E. coli*. Figure S5. Pinene synthase expression in *E. coli* EcpsB by different concentrations of IPTG. SDS-PAGE 4 h after induction.

## Data Availability

All data generated or analyzed during this study are included in this published article and its additional files.
