# Supplementary material for: Metabolic engineering of Deinococcus radiodurans for pinene production from glycerol
Source: Microb Cell Fact. 2021 Sep 26;20:187. doi: 10.1186/s12934-021-01674-4 (PMC8474958; doi:10.1186/s12934-021-01674-4)
Supplement: Supplementary file 1 — Additional file 1: Table S1. List of oligos used for vector construction and real-time PCR. Figure S1. The sequence of the pinene synthase-encoding gene codon-optimized for D. radiodurans.Figure S2. Pinene degradation or consumption by D. radiodurans in 120 h. D. radiodurans was grown in a medium supplemented with 20 mg/L of pinene isomers and residual pinene was quantified by GC-FID after 120 h. Un-inoculated pinene supplemented medium was used as the control. Figure S3. Effect of using different concentrations of dodecane (10% and 20%) a in the rich and b the minimal glycerol medium and various agitation speed (70, 100, 180, and 250 rpm) on the pinene production by D. radiodurans ∆dr0862 P-I-D. Figure S4. GC-FID chromatograms. a Alpha-pinene standard, b beta-pinene standard, c beta-pinene production by D. radiodurans, and d alpha- and beta-pinene production by E. coli. Figure S5. Pinene synthase expression in E. coli EcpsB by different concentrations of IPTG. SDS-PAGE 4 h after induction. [file 12934_2021_1674_MOESM1_ESM.docx]

Additional file 1

Metabolic Engineering of *Deinococcus radiodurans* for Pinene Production from Glycerol

Seyed Hossein Helalat ^1,2^, Carsten Jers ^2^, Mandana Bebahani ^1*^, Hassan Mohabatkar ^1^ , Ivan Mijakovic ^2,3^

^1^Biological science and technology, Isfahan University, Isafahan, Iran

^2^The Novo Nordisk Foundation Center for Biosustainability, Technical University of Denmark, Lyngby, Denmark

^3^Systems and Synthetic Biology Division, Department of Biology and Biological Engineering, Chalmers University of Technology, Gothenburg, Sweden

*** Correspondence:**Mandana Behbahani
[ma.behbahani@ast.ui.ir](mailto:ma.behbahani@ast.ui.ir)

**Table S1**. List of oligos used for vector construction and real-time PCR.

| Primer | Sequence | Description |
| --- | --- | --- |
| Pkat fwd1 | ATATCTCGAGCGTTCCATGAGTCTCCTGTCCCG | *Xho*I |
| Pkat rev1 | ATATTCTAGATTAGATCTTTTCATATGCTCTCCTTCGCCTCGCT | *Nde*I |
| Pgro fwd1 | ATATCTCGAGCATGTTCAGGGATGGAAGCACGTAT | *Xho*I |
| Pgro rev1 | TTATTCTAGATTCATATGGGGTCCTCCTGTGAGTGAGAT | *Nde*I |
| Ptuf fwd1 | ATATCTCGAGACTAGTCTTCGTGACCGTGCCGCTGAT | *Xho*I |
| Ptuf rev1 | ATATGAGCTCCATTGTCTTACTCCCTCCAAGCGG | *Sac*I |
| Ps_fwd | ATATCATATGAGCCGCCGCGGCAAGA | *Nde*I |
| Ps_NS_rev | ATATGGATCCCAGGGGCACGCTTTCCAGCA | *Bam*HI, no stop codon |
| Gfp_fwd | ATTAGGATCCGGCGGTGGCGGCTCTATGGAGAGCGACGAGAGCGG | *Bam*HI*,* linker |
| Gfp_rev | ATTAAAGCTTTTAGCGAGATCCGGTGGAGCC | *Hind*III |
| Pkat fwd2 | ATTAGGATCCGTTCCATGAGTCTCCTGTCCCG | *Bam*HI |
| Pkat rev2 | ATTACCTGCAGGAATATCTAGACATACACTCTCCTTCGCCTCGCTG | *Xba*I |
| Pgro fwd2 | ATTAGGATCCTCATGTTCAGGGATGGAAGCACG | *Bam*HI |
| Pgro rev2 | ATTACCTGCAGGTATATCTAGACATGTGGGGTCCTCCTGTGAGTG | *Xba*I |
| IspA fwd | ATATTCTAGAATGGACTTTCCGCAGCAACTCG | *Xba*I |
| IspA rev | ATATAAGCTTTATTTATTACGCTGGATGATGTAGTCCG | *Hind*III |
| Pkat fwd3 | ATATAAGCTTCGTTCCATGAGTCTCCTGTCCCG | *Hind*III |
| Pkat rev3 | ATTACCTGCAGGATTAGTCGACCATACACTCTCCTTCGCCTCGCTG | *Sal*I |
| Dxs fwd | ATATAAGCTTTATGTCGACATGAACGAACTTCCCGGCACGT | *Sal*I |
| Dxs rev | TAATCCTGCAGGCTACACCTCAATCGGCACGTCCA | *Sda*I |
| Ps(N)_fus fwd | ATATGTCGACATGAGCCGCCGCGGCAAGA | *Sal*I, N-ter PS fusion |
| Ps(N)_fus fwd2 | ATATGAGCTCCAGGGGCACGCTTTCCAGCA | *Sac*I, N-ter PS fusion |
| Isp(C)_fus fwd | ATTAGAGCTCGGTGGTGGTGGTTCTATGGACTTTCCGCAGCAACTCG | *Sac*I C-ter GPPS fusion |
| Isp(C)_fus rev | ATTAGGATCCTTATTTATTACGCTGGATGATGTAGTCCG | *Bam*HI, C-ter GPPS fusion |
| Isp(N)_fus fwd | ATATGTCGACGAATTCATGGACTTTCCGCAGCAACTCG | *Sal*I, N-ter GPPS fusion |
| Isp(N)_fus rev | ATATGAGCTCGGTACCTTTATTACGCTGGATGATGTAGTCCG | *Sac*I, N-ter GPPS fusion |
| Ps(C)_fus fwd | ATATGAGCTCGGTGGTGGTGGTTCTATGAGCCGCCGCGGCAAG | *Sac*I C-ter PS fusion |
| Ps(C)_fus fwd2 | ATTAGGATCCTTACAGGGGCACGCTTTCCAGCA | *Bam*HI, C-ter PS fusion |
| crtB_UP fwd | ATATAGATCTCACCCGCAGGTCCGAATTG | *Bgl*II, 926 bp upstream |
| crtB _UP rev | ATATTCTAGAGCCTAGGCTTCGACACCACCAT | *Xba*I, 926 bp upstream |
| crtB _Dwn fwd | TATAGAATTCCTGCAGGAAAGACAGCACCAGCAAGGC | *Eco*RI, 979 bp downstream |
| crtB _Dwn rev | TATACTCGAGTTGGTCTGGTCGGGGTCGGT | *Xho*I, 979 bp downstream |
| dr1395_UP fwd | ATATAGATCTGGCCTTTTCCAGCGCGTCG | *Bgl*II, 798 bp upstream |
| dr1395_UP rev | ATATTCTAGAATTAGAGCTCCATAGGGGGAGTGTAGTGGGCG | *Xba*I, 798 bp upstream |
| dr1395_Dwn fwd | TATGGATCCCCTGCAGGTATAACTAGTAAGCTTCAAGGACCCCGCCGTCATTG | *Bam*HI, 998 bp downstream |
| dr1395_Dwn rev | ATATCTCGAGCGACCCCTGGACCACCGACA | *Xho*I, 998 bp downstream |
| kanR fwd | TAATCATATGAGCCATATTCAACGGGAAAC | *Nde*I |
| kanR rev | ATTATCTAGATTAGAAAAACTCATCGAGCATCAAAT | *Xba*I |
| Ps-RT fwd | GGGCGACTTCCACAGCAACC | Real time PCR, *ps_DR_* |
| Ps-RT rev | GCTCATCAGTTCGCCGTCTTCC | Real time PCR, *ps_DR_* |
| Gap-RT fwd | GCCAAAGCCGTTGATGCCTACTT | Real time PCR, *dr1343* |
| Gap-RT rev | CGTTGACCGTCAGGCTGCTTTC | Real time PCR, *dr1343* |

| ATGAGCCGCCGCGGCAAGAGCATCACCCCCAGCATCAGCATGAGCAGCACCACCGTGGTGACCGACGACGGCGTGCGCCGCCGCATGGGCGACTTCCACAGCAACCTGTGGGACGACGACGTGATCCAGAGCCTGCCCACCGCCTACGAAGAAAAGAGCTACCTGGAACGCGCCGAAAAGCTGATCGGCGAAGTGAAGAACATGTTCAACAGCATGAGCCTGGAAGACGGCGAACTGATGAGCCCCCTGAACGACCTGATCCAGCGCCTGTGGATCGTGGACAGCCTGGAACGCCTGGGCATCCACCGCCACTTCAAGGACGAAATCAAGAGCGCCCTGGACTACGTGTACAGCTACTGGGGCGAAAACGGCATCGGCTGCGGCCGCGAAAGCGTGGTGACCGACCTGAACAGCACCGCCCTGGGCCTGCGCACCCTGCGCCTGCACGGCTACCCCGTGAGCAGCGACGTGTTCAAGGCCTTCAAGGGCCAGAACGGCCAGTTCAGCTGCAGCGAAAACATCCAGACCGACGAAGAAATCCGCGGCGTGCTGAACCTGTTCCGCGCCAGCCTGATCGCCTTCCCCGGCGAAAAGATCATGGACGAAGCCGAAATCTTCAGCACCAAGTACCTGAAGGAAGCCCTGCAGAAGATCCCCGTGAGCAGCCTGAGCCGCGAAATCGGCGACGTGCTGGAATACGGCTGGCACACCTACCTGCCCCGCCTGGAAGCCCGCAACTACATCCAGGTGTTCGGCCAGGACACCGAAAACACCAAGAGCTACGTGAAGAGCAAGAAGCTGCTGGAACTGGCCAAGCTGGAATTTAACATCTTCCAGAGCCTGCAGAAGCGCGAACTGGAAAGCCTGGTGCGCTGGTGGAAGGAAAGCGGCTTCCCCGAAATGACCTTCTGCCGCCACCGCCACGTGGAATACTACACCCTGGCCAGCTGCATCGCCTTCGAACCCCAGCACAGCGGCTTCCGCCTGGGCTTCGCCAAGACCTGCCACCTGATCACCGTGCTGGACGACATGTACGACACCTTCGGCACCGTGGACGAACTGGAACTGTTCACCGCCACCATGAAGCGCTGGGACCCCAGCAGCATCGACTGCCTGCCCGAATACATGAAGGGCGTGTACATCGCCGTGTACGACACCGTGAACGAAATGGCCCGCGAAGCCGAAGAAGCCCAGGGCCGCGACACCCTGACCTACGCCCGCGAAGCCTGGGAAGCCTACATCGACAGCTACATGCAGGAAGCCCGCTGGATCGCCACCGGCTACCTGCCCAGCTTCGACGAATACTACGAAAACGGCAAGGTGAGCTGCGGCCACCGCATCAGCGCCCTGCAGCCCATCCTGACCATGGACATCCCCTTCCCCGACCACATCCTGAAGGAAGTGGACTTCCCCAGCAAGCTGAACGACCTGGCCTGCGCCATCCTGCGCCTGCGCGGCGACACCCGCTGCTACAAGGCCGACCGCGCCCGCGGCGAAGAAGCCAGCAGCATCAGCTGCTACATGAAGGACAACCCCGGCGTGAGCGAAGAAGACGCCCTGGACCACATCAACGCCATGATCAGCGACGTGATCAAGGGCCTGAACTGGGAACTGCTGAAGCCCGACATCAACGTGCCCATCAGCGCCAAGAAGCACGCCTTCGACATCGCCCGCGCCTTCCACTACGGCTACAAGTACCGCGACGGCTACAGCGTGGCCAACGTGGAAACCAAGAGCCTGGTGACCCGCACCCTGCTGGAAAGCGTGCCCCTGTAA |
| --- |

**Figure S1.** The sequence of the pinene synthase-encoding gene codon-optimized for *D. radiodurans.*


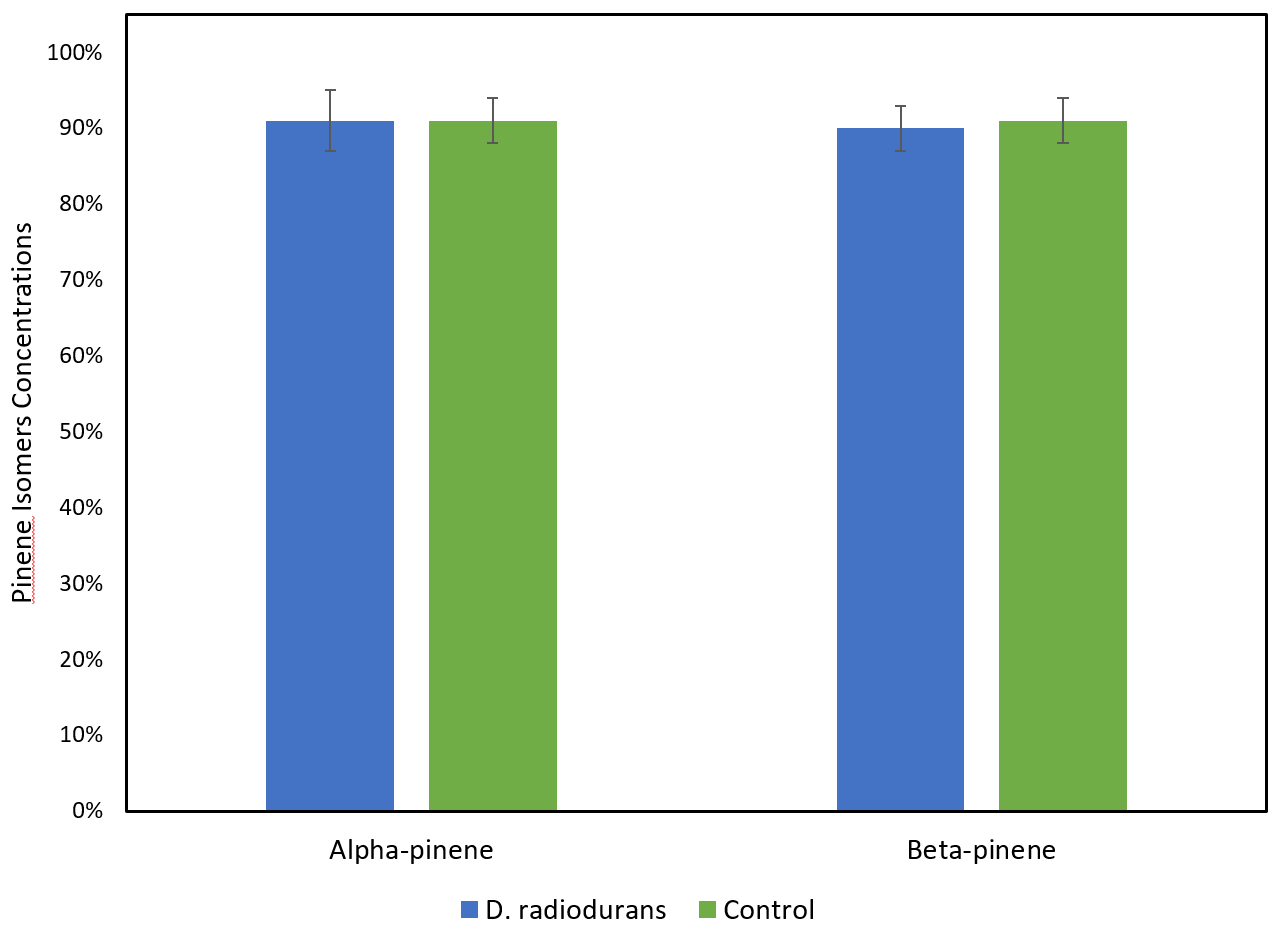


**Figure S2.** Pinene degradation or consumption by *D. radiodurans* in 120 hours. *D. radiodurans* was grown in a medium supplemented with 20 mg/L of pinene isomers and residual pinene was quantified by GC-FID after 120 hours. Un-inoculated pinene supplemented medium was used as the control.


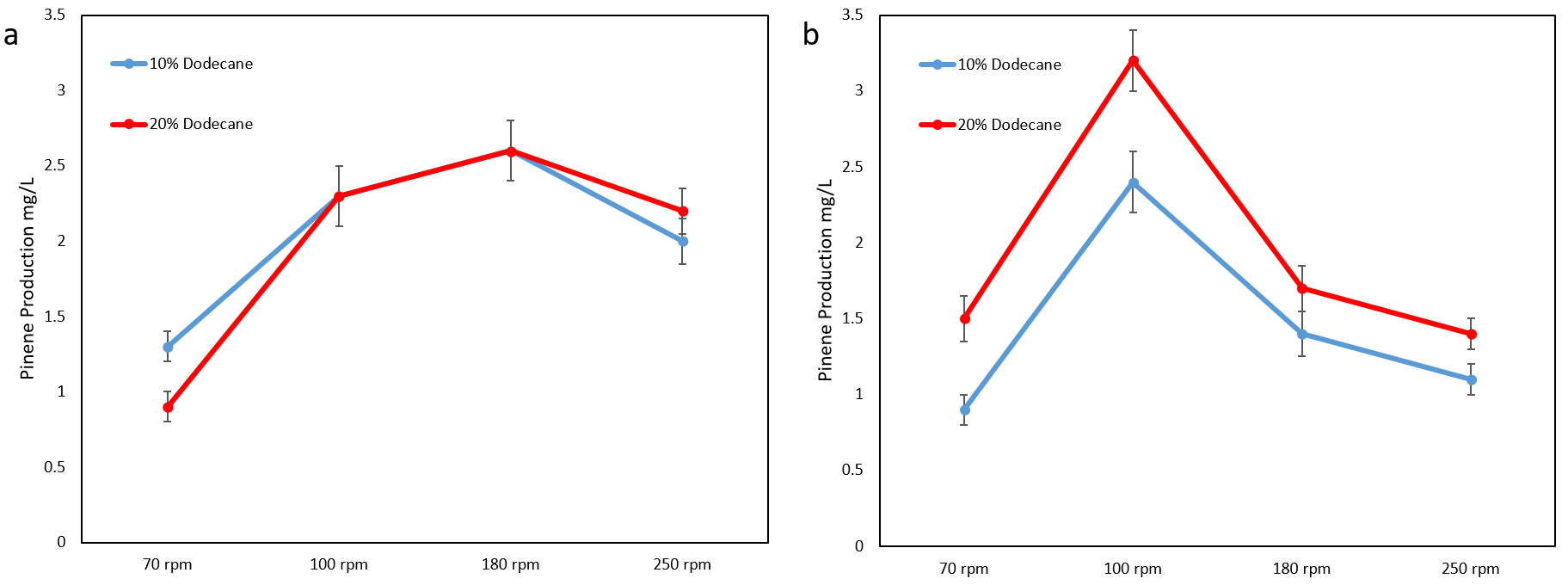


**Figure S3.** Effect of using different concentrations of dodecane (10% and 20%) **a** in the rich and **b** the minimal glycerol medium and various agitation speed (70, 100, 180, and 250 rpm) on the pinene production by *D. radiodurans* ∆*dr0862* P-I-D*.*

**Figure S4.** GC-FID chromatograms. **a** Alpha-pinene standard, **b** beta-pinene standard, **c** beta-pinene production by *D. radiodurans*, and **d** alpha- and beta-pinene production by *E. coli*.


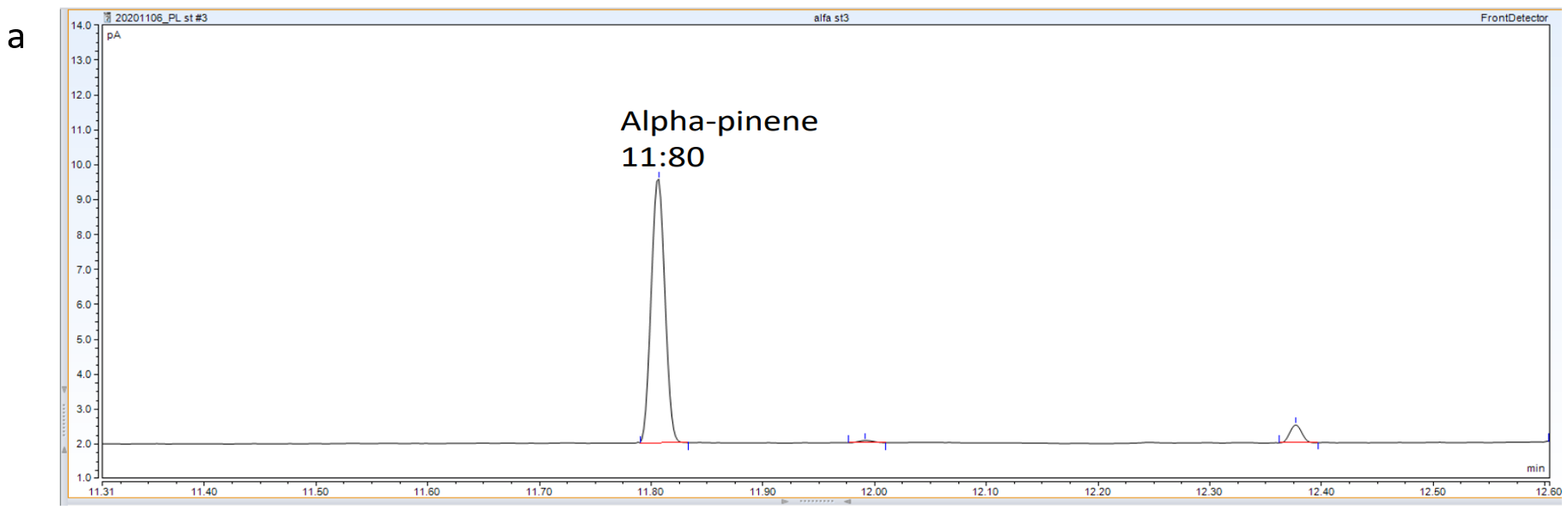

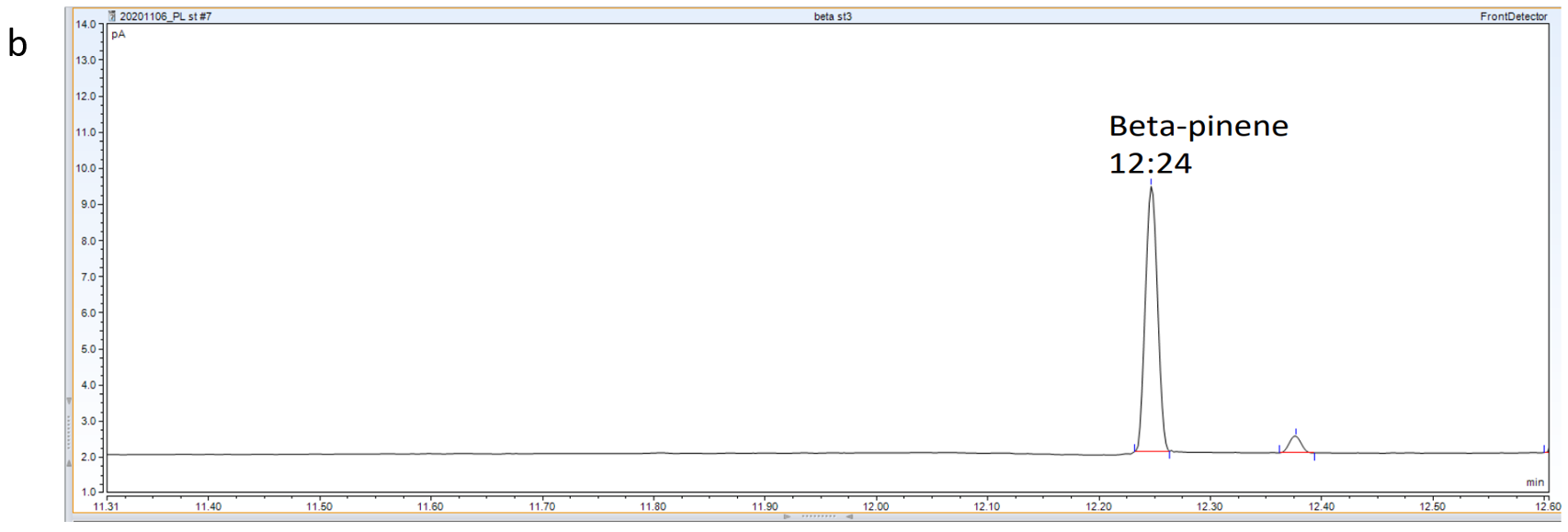

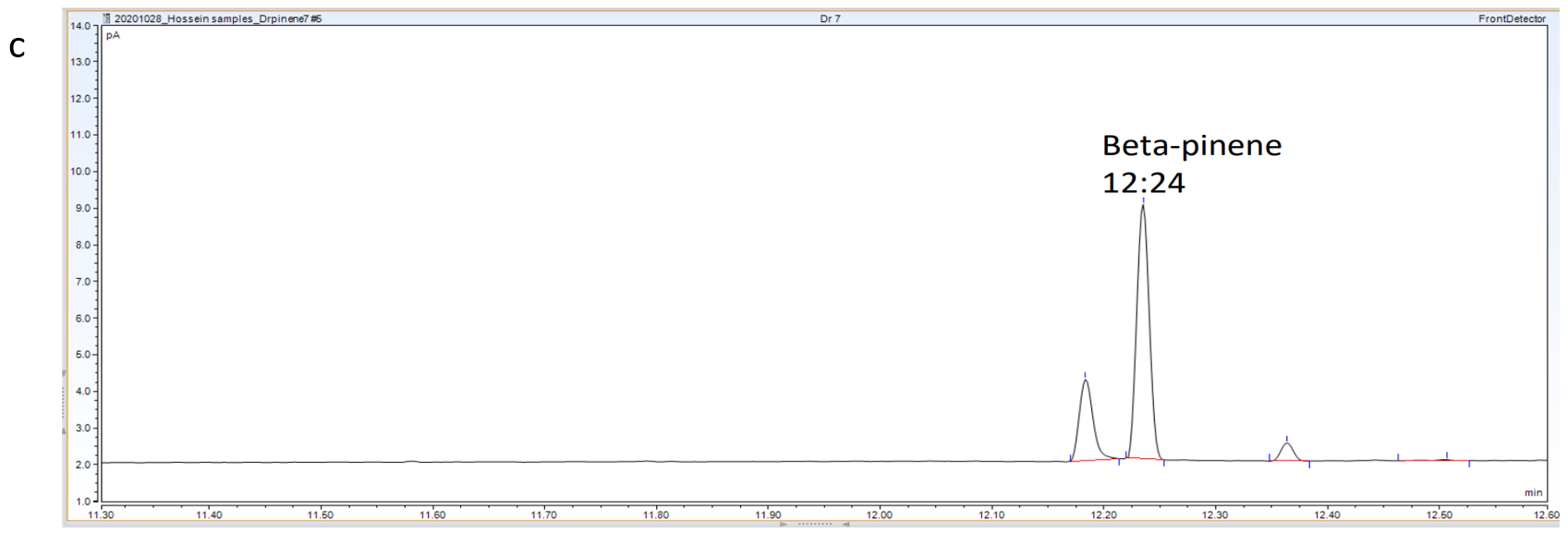

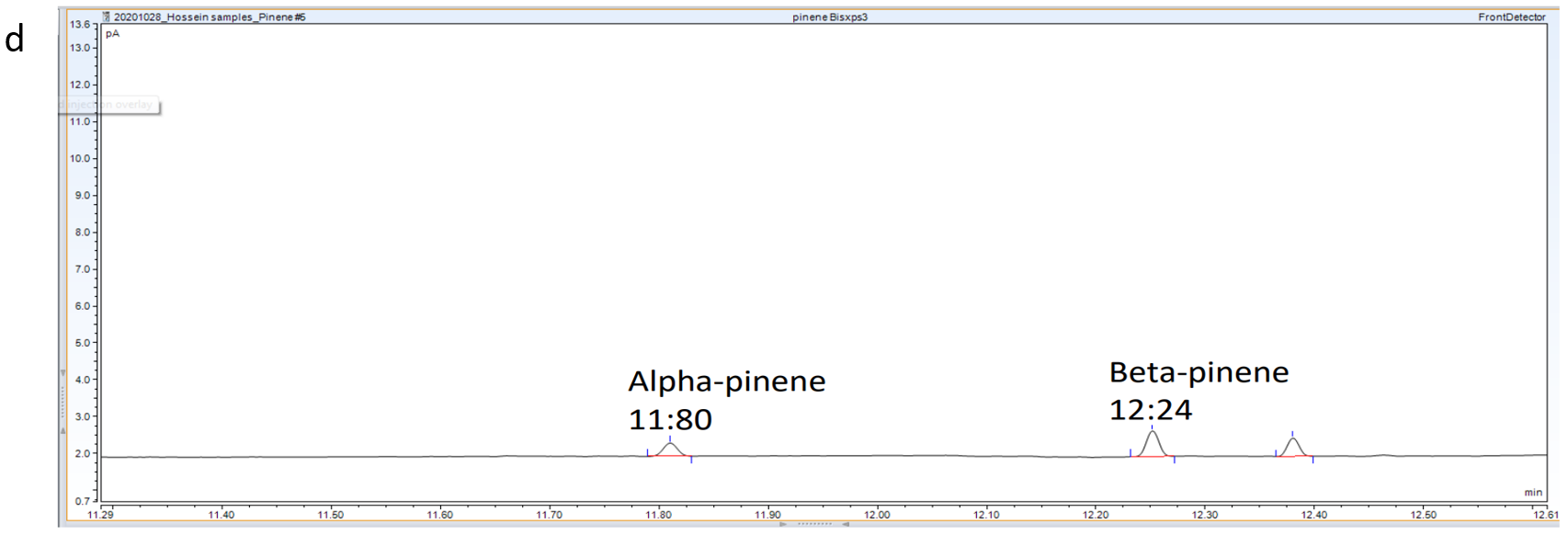


**
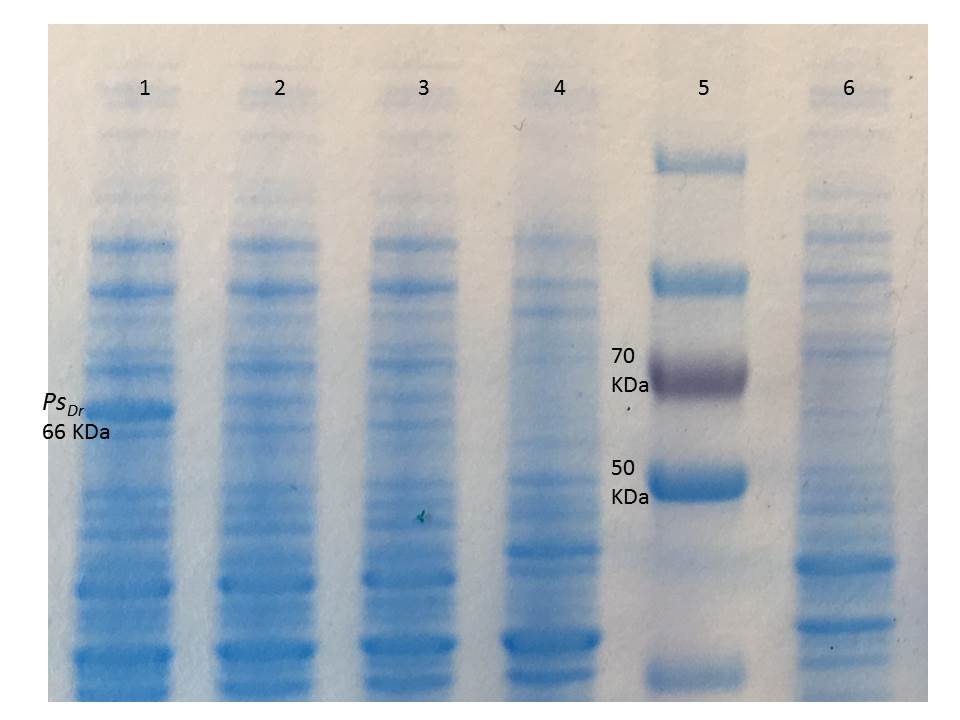
**

**Figure S5.** Pinene synthase expression in *E. coli* EcpsB by different concentrations of IPTG. SDS-PAGE 4 hours after induction. **Lane1** 1 mM, **Lane2** 0.5 mM, **Lane3** 0.1 mM, **Lane4** *E. coli* without plasmids and induction, **Lane5** Protein Ladder, and **Lane6** *E. coli* EcpsB before induction.
